# Early and Late Complications Associated with Penile Cancer Surgery and the Impact of Human Papillomavirus Status: Findings from a Retrospective Norwegian Cohort Study

**DOI:** 10.1016/j.euros.2025.01.009

**Published:** 2025-02-08

**Authors:** Patrick Juliebø-Jones, Ida M. Nordanger, Christian Beisland, Tor K. Thorkelsen, Alfred Honoré, Christian A. Moen

**Affiliations:** a Department of Urology Haukeland University Hospital Bergen Norway; b Department of Clinical Medicine University of Bergen Bergen Norway

**Keywords:** Penile squamous cell carcinoma, Penile intraepithelial neoplasia, Complications, Human papillomavirus

## Abstract

**Background and objective:**

Penile cancer (PeCa) and penile intraepithelial neoplasia (PeIN) are rare diseases, and the burden of complications associated with surgery remains under-reported. The objective was to evaluate the early (≤30 d) and late (>30 d) complications and the impact of human papillomavirus (HPV) status.

**Methods:**

A retrospective analysis was conducted of a cohort consisting of 201 consecutive and treatment-naïve patients with PeCa/PeIN undergoing surgery (penile sparing, and partial and total amputation) between January 1, 2000 and December 31, 2023 at a tertiary centre as part of a centralised regional service.

**Key findings and limitations:**

The median follow-up time was 39 (interquartile range 21, 76) mo. The early and late patient complication rates were 45% and 38%, respectively. Of the patients, 18.5% experienced two or more early complications. A majority (80%) of early complications were minor (Clavien-Dindo ≤2). There was a 1% admission rate to the intensive care unit, but no deaths were recorded within 30 d. Body mass index (BMI)was a significant predictor of early complications (*p* = 0.01). Late complications included chronic wound irritation (10%) and urethral stricture (11%). The latter was highest among those who had undergone partial amputation. One in four patients underwent reoperation due to recurrence during follow-up. HPV status had no association with the rate of either early or late complications.

**Conclusions and clinical implications:**

PeCa surgery is associated with a relatively high complication burden, in both the early and the late postoperative period. Lymph node surgery further adds to the morbidity profile. BMI was a significant predictor of having an early complication, while HPV status did not affect the rate of early or late complications.

**Patient summary:**

Penile cancer surgery is associated with a high rate of complications in early as well as late postoperative period. However, most of these complications are not severe. Body mass index was a significant predictor of an early complication, but human papillomavirus status was not associated with the risk of complications.

## Take Home Message

1. Complications are common among patients undergoing penile cancer surgery.

2. Most complications are not severe.

3. Human papillomavirus status does not affect the risk of complications.

## 1 Introduction

Penile cancer (PeCa) is a rare malignancy and has a reported incidence varying between 0.1 and 2.1 per 100 000 males [1], [2]. For this reason, it is often referred to as an orphan disease [1]. In 2020, an estimated 30 068 new cases were diagnosed worldwide, and a number of epidemiological studies have reported that the global burden of PeCa has increased gradually over time [3], [4]. While there has been a shift towards penile-sparing surgery (PSS), amputative surgery is still indicated in cases of more aggressive disease and, for example, where there is involvement of the cavernosal tissue and/or a large local recurrence [5]. At the time of presentation, approximately 25% of patients have been found to have PeCa that has already reached an advanced stage [6]. There is a recognised morbidity associated with PeCa surgery, and this is especially the case when lymph node (LN) dissection (LND) is performed. While the latter has received increased attention in the current era, the wider profile of surgical complications associated with PeCa surgery remains quite limited [7], [8], [9]. In a recent systematic review by Sood et al [9], the authors identified only two studies with complete reporting on complications after amputative surgery. The lack of data in this regard is also evident when reviewing recent iterations of international guidelines [5], [10]. Available studies often do not differentiate between early and late adverse events when reporting complications [11]. Therefore, the primary aim of this study was to evaluate the burden of early and late complications associated with PeCa surgery. Furthermore, about half of all PeCa tumours are human papillomavirus (HPV) positive [12]. It remains unknown whether HPV status affects the rate of complications following PeCa surgery. The secondary aim was thus to perform exploratory analyses to investigate whether HPV status was associated with the rate of early or late complications following PeCa surgery.

## 2 Patients and methods

### 2.1 Patient selection

As part of the centralised management of PeCa, all cases in Western Norway undergo surgical treatment at Haukeland University Hospital. Upon ethical approval (REK no. 291376), all patients undergoing surgery for PeCa or penile intraepithelial neoplasia (PeIN) at this centre between January 1, 2000 and December 31, 2023 were retrospectively identified. Only four patients had received neoadjuvant chemotherapy, and they were excluded from a further analysis since this number was deemed to be too low for studying the effect of neoadjuvant treatment on the postoperative risk of complications. All patients received written information about the study, including details on how to opt out. Only one patient declined to participate in the study. Electronic records for all the other patients were reviewed to access the relevant patient data. Both in- and outpatient events were eligible for inclusion. In total, a retrospective analysis of the data from 201 treatment-naive patients was performed.

### 2.2 Surgical management of penile procedures

All surgery types for PeCa and PeIN were considered, and these were categorised into the following groups: PSS, partial amputation, and total amputation. Types of PSS included circumcisions, (wide) local excision (WLE), and glans resurfacing procedures where the wound surface was either closed by sutures or reconstructed using a split-thickness skin graft harvested from the thigh. Partial amputations (including glansectomy) were reconstructed by suturing shaft skin to the urethral mucosa. When possible, care was taken to divide the urethra as distal as possible to be able to both spatulate the urethral orifice and achieve a tension-free anastomosis to the skin. When necessary, the margin status was checked by peroperative frozen sections. Of note, larger spatulations such as the Johanson urethroplasty has not been used in this cohort. Total amputations were always reconstructed by the establishment of a perineostomy.

### 2.3 Surgical management of inguinal and pelvic procedures

LN management was categorised as follows: dynamic sentinel node biopsy (DSNB), inguinal LND (ILND), and finally, ILND + pelvic LND (PLND). All LNDs were performed using an open approach. Throughout the study period, the standard of care at our centre has been to perform both penile and inguinal surgery during the primary operation when the patients have clinically node-positive (cN+) disease. Patients with clinically node-negative (cN0) disease were used to be followed by active surveillance. In recent years, however, DSNB has been the standard procedure performed on cN0 groins. At our centre, the sentinel node is sent routinely for intraoperative frozen sectioning, and the penile surgery is performed while waiting for the result of this examination. If frozen sectioning reveals node metastasis, a radical ILND is then performed in the same session.

For cases where radiological examinations have indicated pelvic pathology, we have traditionally also done PLND in the same session if it has been deemed possible to remove all macroscopic tumour tissue during surgery. In line with the current guidelines, however, if there are signs of bulky metastases, we now discuss upfront neoadjuvant chemotherapy before surgery.

### 2.4 Study outcome measures

The primary outcomes of interest were early and late complications. For the early complications, outcomes after the first surgery were noted. For late complications, outcomes after the last surgery, for those operated several times, were noted. All late complications occurring before the end of 2023 were eligible for registration. Early complications were defined as any adverse event occurring ≤30 d after the primary surgery. These were graded according to the Clavien-Dindo (CD) classification system. Complications were also grouped into the following: penile, inguinal, oedema, and other (eg, deep vein thrombosis [DVT]). Complications were defined as late when recorded >30 d postoperatively. All complication types and the corresponding CD grades for the early complications were checked by two experienced urologists to confirm data accuracy.

The secondary outcomes of interest included reoperation rate due to PeCa recurrence. Data were also collected on the baseline demographics, age, body mass index (BMI), and American Society of Anesthesiologists (ASA) physical status among other parameters. In addition, for all included patients, the HPV DNA status of resected tumour tissue stored in the pathology archives was assessed by using the GP5+/GP6+ primer system (Applied Biosystems) for the detection of the HPV *L1* gene, as described in detail elsewhere [13], [14].

### 2.5 Statistical analysis

Quantitative variables were described using median as well as first and third quartiles (Q1 and Q3), while categorical variables were described using frequencies and percentages. Fisher’s exact test was employed to compare complication burdens between different penile surgery types. Multivariable logistic regression with the calculation of individual odds ratios (ORs) and 95% confidence intervals (CIs) was performed as an exploratory analysis to identify any potential risk factors for both early and late complications. The chosen potential risk factors for an early complication were surgical variables (penile and inguinal/pelvic procedures) as well as patient characteristics known from previous studies to be related to the risk of complications after surgery (BMI and ASA score). HPV status was also included to investigate whether HPV infection was related to the development of a complication, after adjustment by the other parameters. For late complications, factors thought to possibly influence the risk of a late complication were included into the analysis. The variables included were late reoperation, type of penile surgery (the most invasive type if operated several times), type of LND (the most invasive type if operated several times), whether adjuvant oncological treatment had been administered, and also HPV status. All statistical tests were performed using R version 4.1.1 [15]. Statistical significance was set at *p* < 0.05.

## 3 Results

### 3.1 Cohort characteristics

Across the 201 patients undergoing surgery for PeCa and PeIN, the median age and BMI were 67 yr and 27, respectively (Table 1). Comorbidities included heart disease (36%), diabetes (14%), and chronic obstructive pulmonary disease (11%). More than half of the patients in the cohort (54%) were either current or ex-smokers. Invasive PeCa was the most common indication for surgery (79%), with PeIN accounting for the remainder (Table 1).

**Table 1:** Summary of patient characteristics

Characteristic	*N* = 201 a
Age	67 (57, 75)
BMI	27 (24, 30)
Indication for treatment	
Invasive cancer	158 (79)
PeIN	43 (21)
Smoking status	
Nonsmoker	92 (46)
Current smoker	54 (27)
Ex-smoker	55 (27)
ASA score	
1	62 (31)
2	91 (45)
3	48 (24)
Heart disease	73 (36)
COPD	23 (11)
Diabetes	28 (14)
Other cancer	41 (20)
HPV status	
HPV negative	97 (48)
HPV positive	104 (52)

ASA = American Society of Anesthesiologists; BMI = body mass index; COPD = chronic obstructive pulmonary disease; HPV = human papillomavirus; PeIN = penile intraepithelial neoplasia; Q = quartile.

a Median (Q1, Q3); *n* (%).

### 3.2 Surgical procedures and HPV types

PSS was performed in 66% of the cases, followed by partial amputation (26%) and total amputation (8.5%; Supplementary Tables 1 and 2). Distribution of these different types of penile surgery when stratified by the tumours’ pathological T stage (pT) is shown in Supplementary Table 3. Of the cohort, 22% underwent ILND and 11% underwent ILND + PLND; 13% underwent sentinel node biopsy (SNB) only, while in 54% of cases, the surgery did not involve LNs. HPV DNA was detected in 104 (52%) of the tumours. Specifically, HPV 16 was present in tumours from 85 patients. HPV 33 was found in seven tumours, while HPV 18 was found in three tumours. HPV 31 and 51 were found in tumours from two patients each. HPV 35, 52, 56, 59, and 73 were found in tumours from one patient each. No patient had more than one HPV type, and all the detected types were classified as high-risk HPV.

### 3.3 Early complications (≤30 d)

Within the first 30 d postoperatively, a total of 146 complications were registered for 91 out of the 201 patients (45%). Of the patients, 18.5% experienced two or more complications. The median (interquartile range [IQR]) hospital stay was 3 (2–7) d (Table 2). The majority (80%) of complications were minor (CD ≤2; Table 2). No deaths were recorded within the first 30 d after PeCa surgery. Two patients required admission to the intensive care unit (CD 4). Complications according to type were as follows: penile 27.5%, inguinal 25.5%, and oedema 15%. In total, sepsis was recorded in seven patients. The overall rate of postoperative bleeding was 1.5%, and this was associated with PSS only. Wound infection was the most common early penile complication (26%), followed by bleeding (1.5%). No significant difference in the rate of penile complications was found between the different surgery types (Table 3).

**Table 2:** Summary of early complications and severity

Characteristic	*N* = 201 a
Hospital stay (d)	3 (2, 7)
Any early complication	91 (45)
Penile complications	
None	145 (72)
Wound infection	53 (26)
Bleeding	3 (1.5)
Inguinal complications	
None	150 (75)
Wound infection	40 (20)
Lymphocele	11 (5.5)
Oedema	
None	171 (85)
Penis/scrotum	19 (9.5)
Penis/scrotum/inguinal	11 (5.5)
DVT	2 (1.0)
Sepsis	7 (3.5)
No. of comp per patient	
0	110 (55)
1	53 (26)
2	23 (11)
3	13 (6.5)
4	2 (1.0)
Clavien-Dindo classification	
Grade 1 complications	120 (60)
Grade 2 complications	41 (20)
Grade 3 complications not under general anaesthesia	23 (11)
Grade 3 complications under general anaesthesia	15 (7.5)
Grade 4 complications requiring ICU management	2 (1.0)
Grade 5 death	0 (0)

comp = complications; DVT = deep vein thrombosis; ICU = intensive care unit.

a Median (Q1, Q3); *n* (%).

**Table 3:** Summary of early and late penile complications according to procedure

	Overall	Penile-sparing surgery	Partial amputation	Total amputation	*p* value a
Early complications	*N_p_* = 201	*N_p_* = 132	*N_p_* = 52	*N_p_* = 17	0.3
None	145 (72)	91 (69)	43 (83)	11 (65)	
Wound infection	53 (26)	38 (29)	9 (17)	6 (35)	
Bleeding	3 (1.5)	3 (2.3)	0 (0)	0 (0)	
Late complications	*N_p_* = 201	*N_p_* = 123	*N_p_* = 57	*N_p_* = 21	0.025
None	158 (79)	99 (80)	44 (77)	15 (71)	
Chronic wound irritation	21 (10)	16 (13)	2 (3.5)	3 (14)	
Urethral stricture	22 (11)	8 (6.5)	11 (19)	3 (14)	

*N_p_* = number of patients in each groups.

Data are presented as *n* (%).

a Fisher's exact test to evaluate whether the distribution of the different complications differed between groups.

Regarding LN surgery, the rate of wound infection was highest (55%) when performing ILND + PLND and lowest for SNB (37%; Table 4).

**Table 4:** Early and late inguinal complications according to the procedure

	Overall (*N* = 201 a)	None (*N* = 108 a)	DSNB (*N* = 27 a)	ILND (*N* = 44 a)	ILND + PLND (*N* = 22 a)
Early complications	*n* = 201	*n* = 108	*n* = 27	*n* = 44	*n* = 22
None	150 (75)	108 (100)	16 (59)	18 (41)	8 (36)
Wound infection	40 (20)	0 (0)	10 (37)	18 (41)	12 (55)
Lymphocele	11 (5.5)	0 (0)	1 (3.7)	8 (18)	2 (9.1)
Late complications	*n* = 201	*n* = 95	*n* = 31	*n* = 50	*n* = 25
None	171 (85)	95 (100)	27 (87)	33 (66)	16 (64)
Chronic scarring	16 (8.0)	0 (0)	2 (6.5)	8 (16)	6 (24)
Lymphocele	14 (7.0)	0 (0)	2 (6.5)	9 (18)	3 (12)

DSNB = dynamic sentinel node biopsy; ILND = inguinal lymph node dissection; PLND = pelvic lymph node dissection.

a Data are presented as *n* (%).

#### 3.3.1 Logistic regression analysis for early complications

This revealed BMI (OR 1.12, 95% CI [1.03, 1.23], *p* = 0.01) as well as any type of inguinal procedure to be a significant predictor of a patient experiencing an early complication (*p* = 0.01; Table 5). Compared with PSS, significantly fewer complications were related to partial amputation (OR 0.25, 95% CI [0.08, 0.71], *p* = 0.01). HPV positivity was not a predictor of an early complication (OR 0.80, 95% CI [0.39, 1.62], *p* = 0.53).

**Table 5:** Logistic regression analysis of predictors of early complications

Characteristic	OR	95% CI	*p* value
BMI	1.12	1.03, 1.23	0.013
Type of penile operation			
Penile-sparing surgery	–	–	
Partial amputation	0.25	0.08, 0.71	0.014
Total amputation	0.28	0.06, 1.23	0.10
LND			
None	–	–	
DSNB	11.1	4.02, 34.1	<0.001
ILND	40.0	12.9, 150	<0.001
ILND + PLND	37.5	9.58, 187	<0.001
ASA score			
1	–	–	
2	0.89	0.39, 2.03	0.78
3+	1.04	0.37, 2.88	0.94
HPV status			
HPV negative	–	–	
HPV positive	0.80	0.39, 1.62	0.53

ASA = American Society of Anesthesiologists; BMI = body mass index; CI = confidence interval; DSNB = dynamic sentinel node biopsy; HPV = human papillomavirus; ILND = inguinal lymph node dissection; LND = lymph node dissection; OR = odds ratio; PLND = pelvic lymph node dissection.

### 3.4 Late complications (>30 d)

In all, 76 (38%) patients experienced a late adverse event. In total, 133 adverse events were recorded. Late penile complications included urethral stricture (11%) and chronic wound irritation (10%). While rates of urethral stricture were highest among patients who had undergone partial amputation (19%), chronic wound irritation was highest in the PSS group (*p* = 0.03; Supplementary Table 5). Of the patients, 15% experienced a late inguinal complication such as chronic scarring (8%) and lymphocele (7%; Supplementary Table 4); 19.5% suffered oedema, with the commonest area affected being the lower extremities (7.5%). Other complications included erysipelas (5.5%), sepsis (4%), and DVT (1%).

#### 3.4.1 Logistic regression analysis for late complications

Having undergone LND or adjuvant treatment in the form of radiation or chemoradiotherapy was a significant predictor of a late complication in logistic regression (Supplementary Table 5). Neither the type of penile surgery, irrespective of whether the patient had been reoperated due to cancer/PeIN recurrence, nor HPV status was a significant predictor of a late complication.

### 3.5 Reoperations/adjuvant treatment due to cancer

The median (IQR) follow-up time was 39 (21, 76) mo. One in four of the patients (25%) underwent repeat surgery due to recurrence of cancer (Supplementary Table 6). This included 4.5% and 2.5% who proceeded to partial and total amputation, respectively. Furthermore, one in four (25%) of the patients received adjuvant oncological treatment during the follow-up period.

## 4 Discussion

This series of 201 patients undergoing PeCa or PeIN surgery at a tertiary centre found that nearly half of the patients experience at least one or more complications within the first 30 d postoperatively. However, only a minority (16%) of these complications are severe in nature, and the mortality rate in this study was zero. In total, 38% experienced a late complication such as urethral stricture (11%) or oedema (19.5%). One in four patients underwent reoperation due to cancer recurrence, and the same fraction of patients received adjuvant oncological treatment during follow-up. Moreover, the study also found that the higher the BMI, the more a patient is at risk of a postoperative complication. HPV status, however, was not associated with the risk of a complication.

This information is valuable when counselling and consenting prospective patients.

Of the few studies reporting on adverse events after PeCa surgery, morbidity rates are not consistent. Li et al [11] published the outcomes of 32 patients who had undergone PSS and reported the complication rate to be only 9.4%. These were all minor in nature; however, none underwent LN surgery [11]. The reported differences are considered to be, at least in part, attributable to a lack of standardised reporting and not just due to differences in surgical technique and/or expertise. For example, in a series of 39 patients undergoing PSS published by Pietrzak et al [16], the reported complications referred to any subsequent revision surgeries due to positive resection margins. In this study, frozen section examination to check for margin status was used frequently. This examination may reduce both future recurrences and the size of the excision, thus minimise overtreatment in PeCa surgery [17]. In the setting of PSS, this term also encompasses laser ablation therapy and techniques such as Mohs micrographic surgery [18]. This means that there is a heterogeneous caseload between studies that renders comparisons difficult, especially when patients undergo combination treatments, for example, laser + WLE or circumcision + topical chemotherapy (5-flurouracil) [19].

Reported complications associated with ILND also vary widely in the literature [20], [21]. A recent survey by Fankhauser et al [22] confirmed how much variation exists regarding the surgical techniques and approaches employed when performing both SNB and ILND. Moreover, an expert consensus on how to report complications in lymphadenectomy of the inguinal area was published recently [23]. Adherence to these recommendations will make it easier to compare studies in the future.

Groin dissection with robotic approaches has been reported in smaller studies, and the findings have revealed shorter hospital stays and lower rates of postoperative oedema and DVT [9]. However, a robotic approach has also been associated with longer operation time and drain duration than, but otherwise similar complication rates to, open surgery [24]. More studies on the optimal technique for a staging procedure are therefore needed. Implementation of lymph flow imaging with agents such as Indocyanine Green has also been proposed as a means to reduce associated complications [20], [25].

Our study identified BMI as a risk factor for early complications. Stuiver et al [26] reported on complications after ILND in a series of 164 patients at the Netherlands Cancer Institute. The authors found that 58% experienced one or more complications, and that BMI was a significant predictor. This is also consistent with the findings from LND studies in melanoma and vulvar cancer patients [27]. One possible explanation for this finding could be that the increased extent of surgery needed to remove more fatty tissue will result in a larger subcutaneous defect that may be more prone to complications. Of note, removal of an increased number of LNs as well as sartorius muscle transposition has also been associated with higher complication rates [20]. Moreover, it is also possible that patients with a higher BMI could be unhealthier and therefore, in general, more at risk of a postoperative complication. Further studies are thus needed to determine the underlying reasons why BMI is a risk factor for complications in PeCa surgery.

Inguinal LN metastasis density has been shown to be lower in patients with HPV-positive disease than in those with HPV-negative disease [28]. However, HPV status was not associated with either early or late complications in this study. In a previous study, the different types of surgery and the types of adjuvant treatment received were similar in distribution for both HPV-positive and HPV-negative patients [29]. That patients undergo similar treatment regimens could therefore explain why HPV status did not affect the investigated rate of complications.

### 4.1 Limitations

This study is limited by its retrospective status and a bias related to elements such as sample selection and surgical indication. Thus, the rate of the different complications may have changed over the study period. For example, over time there has been a shift towards PSS, where not only oncological control, but also life quality issues are taken into consideration [30]. As such, there may be older patients for whom such approaches were not available at the time. At least one experienced PeCa surgeon has been present during all procedures. Therefore, this study did not evaluate learning curves as a possible cause of complications. Furthermore, this study did not capture subjective outcomes, for example, quality of life and sexual function, such as through the delivery of patient-reported outcome measures [31]. Future studies should aim to assess all these elements and in a prospective format. Given that the standard of care in our centre is to perform same-session penile and inguinal surgery, when indicated, the complications for both these types of surgery have been pooled. In addition, practice patterns vary in this regard, and therefore our findings may be less generalisable in settings where these are performed on a staged basis. Logistic regression to identify separate risk factors for penile and LN surgery was therefore also not possible.

There were too few patients who had received neoadjuvant treatment in our cohort to be able to reasonably study the effect of preoperative chemotherapy on postoperative complications in the present study. This should therefore be studied further in future investigations. The risk of an early complication was highly associated with having performed LND. A more robust estimate of the exact risk of an early complication after LND could have been obtained by including more patients in the analysis.

## 5 Conclusions

A relatively high number of patients undergoing PeCa surgery experience at least one early complication, if not more. However, the majority of these are minor in nature. A similar but slightly lower proportion of patients experience a late complication. BMI was determined to be a significant predictor of having an early complication. Morbidity is increased when the patient also undergoes LND. One in four patients underwent reoperation due to PeCa recurrence during the follow-up period. HPV status was not associated with either early or late complications.

  ***Author contributions*:** Patrick Juliebø-Jones had full access to all the data in the study and takes responsibility for the integrity of the data and the accuracy of the data analysis.

  *Study concept and design*: Juliebø-Jones, Beisland, Moen.

*Acquisition of data*: Nordanger, Honoré, Thorkelsen, Moen.

*Analysis and interpretation of data*: Juliebø-Jones, Nordanger, Honoré, Thorkelsen, Beisland, Moen.

*Drafting of the manuscript*: Juliebø-Jones, Beisland, Moen.

*Critical revision of the manuscript for important intellectual content*: Juliebø-Jones, Nordanger, Honoré, Thorkelsen, Beisland, Moen.

*Statistical analysis*: Moen.

*Obtaining funding*: None.

*Administrative, technical, or material support*: None.

*Supervision*: Moen, Beisland.

*Other*: None.

  ***Financial disclosures:*** Patrick Juliebø-Jones certifies that all conflicts of interest, including specific financial interests and relationships and affiliations relevant to the subject matter or materials discussed in the manuscript (eg, employment/affiliation, grants or funding, consultancies, honoraria, stock ownership or options, expert testimony, royalties, or patents filed, received, or pending), are the following: None.

  ***Funding/Support and role of the sponsor*:** The Norwegian Institute of Urology provided financial support for expenses regarding the analysis of HPV DNA status on archival tissue. This institution did not play any part in the development or the approval of this manuscript.

  ***Ethics statement*:** This study was approved by the ethical committee (REK no. 291376).
