# Supplementary material for: Early and Late Complications Associated with Penile Cancer Surgery and the Impact of Human Papillomavirus Status: Findings from a Retrospective Norwegian Cohort Study
Source: Eur Urol Open Sci. 2025 Feb 8;72:29–35. doi: 10.1016/j.euros.2025.01.009 (PMC11849643; doi:10.1016/j.euros.2025.01.009)
Supplement: Supplementary Data 1 [file mmc1.docx]

| **Variable** | **PeIN**  N = 43^1^ | **Penile cancer**  N = 158^1^ |
| --- | --- | --- |
| **Type of Operation** |  |  |
| Penile sparing surgery | 40 (93%) | 92 (58%) |
| Partial amputation | 3 (7.0%) | 49 (31%) |
| Total amputation | 0 (0%) | 17 (11%) |
| **Type of procedures** |  |  |
| Circumcision | 12 (28%) | 16 (10%) |
| Local excision | 16 (37%) | 25 (16%) |
| Wide local excision/resurfacing | 12 (28%) | 50 (32%) |
| Glansectomy | 3 (7.0%) | 26 (16%) |
| Partial amputation | 0 (0%) | 24 (15%) |
| Total amputation | 0 (0%) | 17 (11%) |
| **Frozen sectioning** | 5 (12%) | 67 (42%) |
| **Split-thickness skin graft** | 7 (16%) | 22 (14%) |
| ^1^n (%) | | |

Supplementary Table 1. Breakdown of surgery/procedure type

| **Characteristic** | **Number of patients**  **N = 201**^1^ | **Number of patients with any complication**  **N = 91^1^** |  |
| --- | --- | --- | --- |
| **Type of surgical procedure** |  |  |  |
| PSS | 90 (45%) | 19 (21%) |  |
| PSS + DSNB | 20 (10.0%) | 15 (75%) |  |
| PSS + ILND | 14 (7.0%) | 14 (100%) |  |
| PSS + ILND + PLND | 8 (4.0%) | 8 (100%) |  |
| PA | 13 (6.5%) | 2 (15%) |  |
| PA + DSNB | 7 (3.5%) | 3 (43%) |  |
| PA + ILND | 25 (12%) | 16 (64%) |  |
| PA + ILND + PLND | 7 (3.5%) | 5 (71%) |  |
| TA | 4 (2.0%) | 0 (0%) |  |
| TA + DSNB | 1 (0.5%) | 1 (100%) |  |
| TA + ILND | 5 (2.5%) | 4 (80%) |  |
| TA + ILND + PLND | 7 (3.5%) | 4 (57%) |  |
| ^1^n (%). DSNB = Dynamic sentinel node biopsy, ILND = Inguinal lymph node dissection, PA = partial amputation, PLND = Pelvic lymph node dissection, PSS = Penile sparing surgery, TA = Total amputation | | |  |

Supplementary Table 2. Summary of main surgical procedures and complications

|  | **PSS**  N = 132^1^ | **Partial amputation**  N = 52^1^ | **Total amputation**  N = 17^1^ |
| --- | --- | --- | --- |
| **pT-stage** |  |  |  |
| PeIN | 41 (31%) | 3 (5.8%) | 0 (0%) |
| T1 | 72 (55%) | 13 (25%) | 1 (5.9%) |
| T2 | 17 (13%) | 24 (46%) | 2 (12%) |
| T3 | 2 (1.5%) | 12 (23%) | 11 (65%) |
| T4 | 0 (0%) | 0 (0%) | 3 (18%) |
| ^1^n (%) | | | |

Supplementary Table 3. T stage against penile operation type

| **Characteristic** | **N = 201**^1^ |
| --- | --- |
| **Any late complication** | 76 (38%) |
| **Penile complications** |  |
| None | 158 (79%) |
| Chronic wound irritation | 21 (10%) |
| Urethral stricture | 22 (11%) |
| **Inguinal complications** |  |
| None | 171 (85%) |
| Chronic scarring | 16 (8.0%) |
| Lymphocele | 14 (7.0%) |
| **Oedema** |  |
| None | 162 (81%) |
| Oedema legs | 15 (7.5%) |
| Oedema penis/scrotum | 12 (6.0%) |
| Oedema penis/scrotum and legs | 12 (6.0%) |
| **DVT** | 2 (1.0%) |
| **Erysipelas** | 11 (5.5%) |
| **Sepsis** | 8 (4.0%) |
| ^1^n (%) | |

Supplementary Table 4. Summary of late complications

| **Characteristic** | **OR**^1^ | **95% CI**^1^ | **p-value** |
| --- | --- | --- | --- |
| **Reoperated** |  |  |  |
| No | — | — |  |
| Yes | 1.51 | 0.67, 3.37 | 0.31 |
| **Type of penile surgery** |  |  |  |
| Penile sparing surgery | — | — |  |
| Partial amputation | 0.72 | 0.28, 1.72 | 0.47 |
| Total amputation | 0.38 | 0.08, 1.51 | 0.19 |
| **LND** |  |  |  |
| None | — | — |  |
| DSNB | 1.87 | 0.66, 5.05 | 0.22 |
| ILND | 4.55 | 1.76, 12.2 | 0.002 |
| ILND + PLND | 8.87 | 1.96, 46.4 | 0.006 |
| **Adjuvant** |  |  |  |
| No adjuvant treatment | — | — |  |
| Chemotherapy | 0.72 | 0.10, 3.37 | 0.70 |
| Radiation | 5.04 | 1.42, 21.2 | 0.017 |
| Chemoradiotherapy | 12.3 | 2.52, 96.5 | 0.005 |
| **HPV status** |  |  |  |
| HPV negative | — | — |  |
| HPV positive | 0.59 | 0.28, 1.20 | 0.15 |
| ^1^OR = Odds Ratio, CI = Confidence Interval. DSNB = Dynamic Sentinel Node Biopsy; HPV = Human Papillomavirus; ILND = Inguinal Lymph Node Dissection; PLND = Pelvic Lymph Node Dissection | | | |

Supplementary Table 5. Logistic regression analysis of predictors of a late complication.

| **Characteristic** | **N = 201**^1^ |
| --- | --- |
| **Reoperation due to cancer** | 50 (25%) |
| **Reop penile surgery** |  |
| No | 157 (78%) |
| Penile sparing surgery | 30 (15%) |
| Partial amputation | 9 (4.5%) |
| Total amputation | 5 (2.5%) |
| **Reop lymph node surgery** |  |
| No | 185 (92%) |
| DSNB | 3 (1.5%) |
| ILND | 10 (5.0%) |
| ILND + PLND | 3 (1.5%) |
| **Adjuvant therapy** |  |
| No adjuvant treatment | 151 (75%) |
| Chemotherapy | 10 (5.0%) |
| Radiation | 21 (10%) |
| Chemoradiotherapy | 19 (9.5%) |
| **Follow up time (Months)** | 39 (21, 76) |
| ^1^n (%); Median (Q1, Q3). Q = Quartile. | |

Supplementary Table 6. Summary of reoperations due to cancer
